# Validity of heart rate derived core temperature estimation during simulated firefighting tasks

**DOI:** 10.1038/s41598-023-49929-x

**Published:** 2023-12-15

**Authors:** Philip J. Agostinelli, Braxton A. Linder, Katherine A. Frick, Nicholas C. Bordonie, Frances K. Neal, JoEllen M. Sefton

**Affiliations:** 1 Warrior Research Center, School of Kinesiology, Auburn University, 301 Wire Road, Auburn, AL 38632 USA; 2 Neurovascular Physiology Lab, School of Kinesiology, Auburn University, 301 Wire Road, Auburn, AL USA

**Keywords:** Physiology, Health care, Risk factors

## Abstract

Rectal core temperature monitoring can help fire services mitigate heat injury but can be invasive and impractical. EQ02 + LifeMonitor provides a non-invasive estimation of core temperature. Therefore, the primary purpose of this study was to determine the validity of the EQ02 + LifeMonitor compared to the gold standard rectal thermometer core temperature assessment, as well as the potential influence of turnout gear on the estimated and physiological strain experienced during these activities. Thirteen participants completed simulated firefighting tasks with and without turnout gear, involving four rounds of a 5-min walk on a treadmill at 2.8 mph/2.5% grade and 20 deadlifts over 5 min in an environmental chamber set to 40.6 °C; 50% humidity. During each trial participants wore both an EQ02 + LifeMonitor and DataTherm II rectal thermometer. The results from the devices were statistically equivalent (*p* < 0.001), yet there was a statistically significant difference in the value (~ 0.1 °C; *p* < 0.001). There was a significant effect of devices [*p* < 0.001] and time [*p* < 0.001], but no interaction effect [*p* = 0.70] on core temperature drift. Estimated core temperature was marginally different from that measured via the DataTherm II. The EQ02 on average overestimated core temperature. Heart rate, rating of perceived exertion, and area under the curve of core temperature were significantly elevated due to turnout gear [*ps* < 0.025], but not core temperature skin temperature, or ventilatory rate [*ps* > 0.372]. These results suggest the EQ02 + LifeMonitor may be a viable, non-invasive alternative for assessing core temperature compared to rectal temperature monitoring, especially during rigorous, intermittent activities. Turnout gear does however increase heart rate, cumulative core temperature, and perceived exertion. Additionally, the validity of the estimated core temperature is not impacted by the use turnout gear. This is likely due to significant changes in heart rate, which allowed the heart-rate derived estimate of core temperature to remain consistent with changes in DataTherm II rectal temperatures.

## Introduction

Firefighters are frequently exposed to heat stress. A recent study indicates ~ 75% of firefighters have experienced heat-injury related symptoms with 5% experiencing these symptoms more than 20 times in a year^1^. Firefighter research often involves the use of heated environments^2,3^ and high levels of activity due to required occupational demands^4–9^. The necessary personal protective equipment (PPE), while life protecting, further increases heat load and exertional stress due to its insulative properties^1,10–13^. Research has shown firefighters in these environments experience core temperature increases to 39 °C or higher^13^. Heat related injury can occur as core body temperature approaches 40 °C^1^. Thus, real time assessments of core temperature remain vital in firefighter workload monitoring and heat related injury research^14^.

The current standard of core temperature assessment in scientific research and emergency medical settings involves using a rectal thermometer^15,16^. Other methods include gastrointestinal pills, tympanic temperatures, and esophageal temperatures^17^. However, these methods are highly invasive or often impractical for daily field usage^18^. Recent efforts to develop non-invasive core temperature monitoring systems include the Equivital EQ02 + LifeMonitor and Black Ghost software, referred to herein as Equivital. This system is comprised of a physiological monitoring strap and validated heart rate-based algorithm to obtain an estimated core temperature^19–21^. The initial validation of the algorithm was completed with a fairly homogenous sample of healthy young US military volunteers in live-field environments to generate the core temperature estimate model^20^. Subsequent studies validating the model in both controlled and live-field environments involving activities such as treadmill and cycling exercise or ruck marching. These activities are generally classified as continuous steady state exercise^22^. However, firefighting requires repeated stop-and-go type activities^23^. Previous validation attempts suggest that the estimated core temperature provided by the Equivital was not a viable option for monitoring core temperature during live fire training activities when compared to ingestible thermometer pills^24^. Ingestible pills are an accepted tool for in-the-field core temperature monitoring although they are expensive and come with their own challenges. Rectal temperature monitoring remains the gold standard in core temperature monitoring. This presents a potential flaw in validating the Equivital system as the Lin’s Concordance Correlation Coefficient test is intended to validate an experimental measure to the gold-standard^23^.

The primary aim of this study was to assess the validity of a heart rate derived estimation of core temperature compared to rectal thermometer temperatures during simulated firefighting tasks. Our primary hypothesis was there would be high agreement between the rectal temperature measures and Equivital estimates. This was assessed using two one-sided *t*-tests for an equivalence test between the devices. Our secondary aim investigated potential discrepancies in core temperature drift between continuous rectal temperature measures and the Equivital core temperature estimates. The secondary hypothesis is that differences would be evident in temperature drift trajectories between the rectal core temperature measurement and Equivital estimated core temperature through the continuous exercise task. This was assessed as average core temperature from each device over 5-min epochs. The tertiary hypothesis was turnout gear would significantly increase physiological strain and significantly increase ratings of perceived exertion. The impact of turnout gear on physiological strain (core temperature, heart rate, ventilatory rate, and skin temperature responses), and rate of perceived exertion (RPE) experienced during experimental bouts of simulated occupational activities were also evaluated. Finally, our exploratory aim included the use area under the curve (AUC) as a potential, novel, method of quantifying heat stress with respect to both changes in core body temperature and time of exposure. To assess this, the differences in AUC heat stress from paired recreation clothing and firefighter turnout gear exercise trials as measured by the gold-standard rectal core temperature monitoring.

## Methods

### Participants

A total of 13 participants, eight males and five females, were recruited for this study. All participants were individuals from the local community and met the following inclusion criteria: 19–45 years old and comfortable and familiar carrying out exercise tasks while in an environmental chamber. These criteria were in place to closely match our target population of firefighters while ensuring foundations of fitness were met for the required simulated tasks. Exclusion criteria included: a known medical condition preventing their participation in exercise or requiring medically supervised exercise, currently rehabilitating from recent musculoskeletal injury, currently taking anticoagulants, a history of a heart condition or high blood pressure, angina during exercise, recent heat related injury or orthopedic injury, or currently pregnant. A health history questionnaire and informed consent was completed prior to their engagement in any research activity. All study protocols were approved by the Institutional Review Board of Auburn University (protocol code #22-149).

### Research design

This was a randomized cross-over design study. Participants completed simulated firefighter tasks in an environmental chamber on two separate occasions. Visits were identical except for clothing condition [i.e., recreational exercise clothes (t-shirt and shorts) vs the addition of firefighter turnout gear (bunker jacket and pants)]. Block randomization was generated in Microsoft^®^ Excel (Microsoft Corporations, Redmond, WA). A 7-day wash-out period was included between trials to reduce the risk of residual fatigue impacting subsequent trial performance.

### Experimental trials

Baseline measurements of blood pressure, height, weight, and hydration status were assessed prior to completing the simulated firefighter tasks (SFT). Participants were then fitted with an Equivital EQ02 + LifeMonitor (Hidalgo, Cambridge, UK) and a DataTherm^®^ II Temperature Monitor 2M Probe Sensor (RG Medical Diagnostics, Wixom, MI, USA) to collect physiological responses during SFT. The Equivital Black Ghost software was used to estimate core temperature during the SFT (Hidalgo, Cambridge, UK). SFT were conducted in an environmental chamber (ESPEC North America, Inc., Hudsonville, MI, USA) during both conditions to control the temperature and humidity at a constant setting of 40.6 °C (105 °F) and 50% relative humidity. The temperature was chosen to replicate non-fireground activities such as salvage, highway extractions, and other hot summer emergency responses^25–27^. Environmental chamber conditions were monitored throughout testing to assure a consistent temperature and humidity was maintained during trials. Participants were asked to maintain consistent dietary and exercise habits throughout the study. Participants provided a urine sample to ensure adequate hydration prior to each trial for via urine specific gravity (USG) by portable refractometer (V-Resourcing, Hunan, China). Adequate hydration was defined as a USG value less than 1.025 based on the standard set by the National Athletic Trainers’ Association^28^. Additionally, participants were asked to refrain from caffeine, alcohol, and vigorous exercise for 24-h leading up to the trials.

#### Simulated firefighting tasks (SFT)

The SFT included 5 min of deadlifts (one repetition every 15 s) with 40% of participants’ body weight, followed by 5 min of treadmill walking set at 4.5 kph and 2.5% grade. This process was repeated for a total of four rounds (40 min of exercise). Intensities were chosen based on discussion with leadership of the local fire departments and reported average physical demands required by firefighters as established by previous research and the National Fire Protection Association (NFPA)^5,29^. Termination criteria included: the participants reaching volitional fatigue or a rectal core temperature of 39.4 °C. If participants reached 38.9 °C, exercise was terminated, and the participant remained in the chamber for the remainder of the prescribed time. If core temperature continued to rise and reached 39.4 °C participants were removed from the chamber immediately. This termination criteria was set to reduce the risk of exertional heat illness which typically occurs at temperatures > 40 °C^30^.

#### Physiological strain measures

Variables acquired by the Equivital included heart rate, respiratory rate, skin temperature, and estimated core temperature. However, as respiratory rate is specifically a measure of gas exchange, we will herein be referring to this measure as ventilatory rate because the Equivital uses an estimation of mechanical inspiration and expiration (breathes/minutes). Skin temperature was measured at the torso by the Equivital. and is measured by an infrared temperature sensor on the back of the Equivital device in contact with the individual’s skin, under the individual's arm (at the mid-axillary line of the thorax in line with individual’s xiphoid process of the sternum). Skin temperature was recorded every minute during SFT. Core temperature was monitored continuously by a DataTherm^®^ II Temperature Monitor 2M Probe Sensor (RG Medical Diagnostics, Wixom, MI, USA). The DataTherm rectal thermometer was inserted approximately 15 cm into the rectal cavity by the participant. This depth was chosen based on previous research that determined this depth provides the best measure of rectal core temperature^31^. The rectal thermometer was permanently marked at 15 cm for participants to assure they inserted to the correct length. Participants then secured the remainder of the rectal thermometer with adhesive tape to secure the device and reduce movement of the probe during the protocol. For confirmation, participants were asked if the thermometer had been inserted to the marked line prior to visit continuation. After assurance of probe insertion length, participants entered the heat chamber to complete SFT. Rate of perceived exertion (RPE) was assessed every 5 min utilizing the Borg 6–20 scale^32,33^ to understand perceptual effects of the SFT and gear.

### Statistical analysis

All outcome variables were assessed for normality using the Shapiro–Wilk test and visual inspection of Q–Q plots. To determine equivalence, a two one-sided paired samples *t*-test (TOST)^34,35^ was run to determine equivalence between Equivital and DataTherm assessments. Additionally, a Bland–Altman Plot was utilized including equivalence bounds set at mean differences of ± 0.5 °C. Equivalence bounds were determined using a combination of previous research studies^20,36,37^. Specifically, acceptable error ranged from ± 0.40 °C in cyclists to ± 0.5 to ± 0.63 °C in tactical athletes^20,36,37^. Lin’s Concordance Correlation (LCC) was used to determine agreement between the Equivital estimated core temperature and measured DataTherm rectal core temperatures assessment.

Equivital estimated core temperature and DataTherm measurements were averaged over 5-min epochs to compare differences in core temperature drift between devices. The first epoch (i.e., minutes 1–5) was used as the origin and was subtracted from all subsequent epochs to normalize changes in core body temperature to each participant’s trial day baseline. These 5-min core temperature epochs were analyzed using a three-way repeated measures ANOVA (device × time × condition) to determine any differences in temperature drift between the two devices across both conditions. Finally, to compare the effects of wearing the firefighter turnout gear against recreational clothing we ran a MANOVA (time × clothing) to assess multivariate differences of physiological data measured by the Equivital (estimated core temperature, skin temperature, heart rate, and ventilatory rate). Five-minute averages were calculated for these physiological variables under each condition (i.e., recreational clothing or the fire fighter turnout gear) and RPE was collected at the end of each 5-min segment. An ANOVA was also completed to investigate the potential differences in rating of perceived exertion (RPE) induced by turnout gear. Previous research indicating cumulative heat exposure in addition to maximal core temperature^38^ prompted an exploratory aim in which we calculated area under the curve (AUC) to assess total heat exposure. The use of AUC as a metric of cumulative heat strain experienced accounts for the temperature reached and the time spent at elevated core temperatures. Five-minute change scores underwent trapezoidal summation to calculate total AUC. Student’s *t*-test was used to compare potential AUC heat stress between recreation clothing or firefighter turnout gear under identical exercise and environmental conditions. Additionally, the environmental chamber temperature and humidity was monitored continuously throughout the protocol and values were recorded every 5-min to ensure accurate environmental conditions. All significance was set a priori to *p* < 0.05. Statistics were run in jamovi 2.0.0.0^39^ and R Studio 4.1.0^40^.

### Ethical approval

The study was conducted in accordance with the Declaration of Helsinki and approved by the Institutional Review Board (or Ethics Committee) of Auburn University (protocol code #22-149 MR 2205, 05/05/2022).

### Consent to participate

Informed consent was obtained from all individual participants included in the study.

## Results

### Descriptive statistics

Thirteen individuals (Males = 8 Females = 5; Age: 27.1 ± 5.0 years; Height: 172.5 ± 8.7 cm; Weight: 80.1 ± 11.7 kg) were included in analysis. Physiological responses (core temperature means and maximum values) to the SFT are included in Table 1. The average environmental temperature was 40.5 ± 0.1 °C and humidity was 51 ± 4%.

**Table 1:** Physiological strain average and maximal values.

Variable	Recreational clothing (mean; max)	Turnout gear (mean; max)
Equivital core temperature (°C)	37.71 ± 0.30; 38.08 ± 0.36	37.89 ± 0.26; 38.46 ± 0.38
Rectal core temperature (°C)	37.62 ± 0.24; 38.02 ± 0.36	37.81 ± 0.26; 38.42 ± 0.24
Heart rate (bpm)	126.83 ± 15.26; 146.00 ± 16.40	141.09 ± 15.50; 163.54 ± 18.21
Ventilatory rate (breaths/min)	25.44 ± 3.77; 36.15 ± 6.97	26.86 ± 4.33; 38.69 ± 9.58
Skin temperature (°C)	36.93 ± 0.83; 38.29 ± 0.62	37.08 ± 0.79; 37.42 ± 8.31
Rating of perceived exertion	9.57 ± 1.78; 11.31 ± 2.25	11.23 ± 2.01; 18.00 ± 3.07

Values reported as mean ± standard deviation during the 40-min simulated firefighting tasks. Average: The average value across both conditions. Borg 6–20 RPE scale was used of Rating of Perceived Exertion.

### Equivital core temperature validation against DataTherm

**Figure 1:**
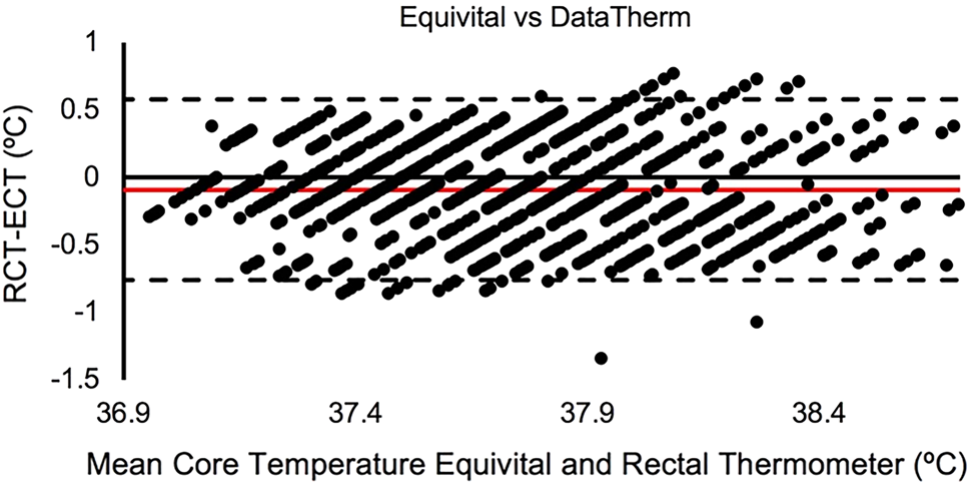
Bland–Altman plot comparing estimated core temperature of the Equivital (ECT) to the measured rectal core temperature (RCT). Dotted lines = Upper and Lower limits of agreement. Red Line = Bias Mean difference reported as degrees Celsius. Made in Microsoft Excel.

**Figure 2:**
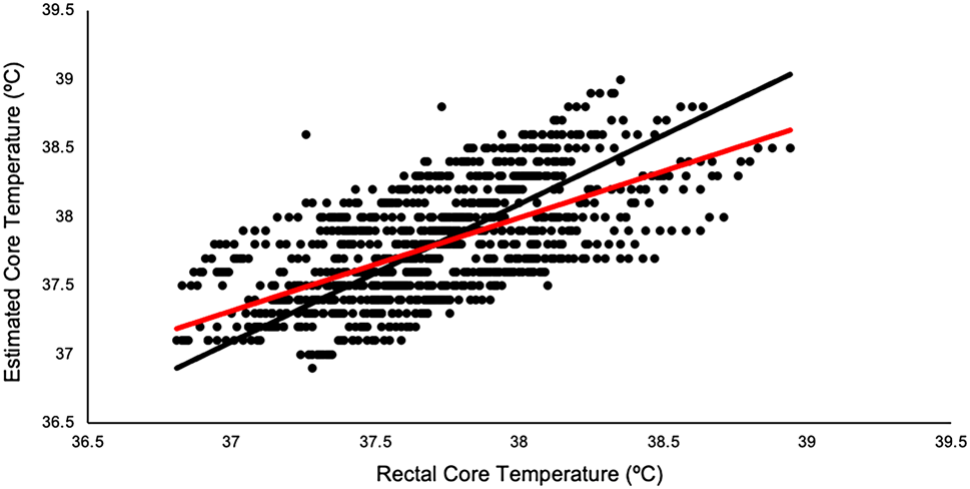
Lin’s concordance correlation to determine the agreement between the Equivital and DataTherm. Made in Microsoft Excel. Red line = actual line LCC; Black line = 1.0 correlation for comparison.

One thousand and six paired data points were generated across the thirteen participants for the validation analysis. Our equivalence test indicated a significant bias of 0.1 °C towards the Equivital overestimating rectal core body temperature measures. Although the Equivital was found to be significantly different from the rectal thermometer, the TOST also found the two measures to be statistically equivalent (Δ_upper_ *p* < 0.001, Δ_lower_ *p* < 0.001; Estimate = − 0.092, 90% CI = − 0.074 to 0.109). The Bland–Altman plot (Fig. 1) suggests a bias: − 0.092, 95% Confidence Interval [− 0.113, − 0.071], Lower Limits of Agreement: − 0.761, 90% CI [− 0.785, − 0.737], and Upper Limits of Agreement: 0.578, 90% CI [0.554, 0.601]). LCC (Fig. 2) determined good concordance^41^ between the Equivital core temperature estimation and DataTherm assessment (LCC = 0.623, 95% CI [0.585, 0.658]).

### Core temperature drift between the Equivital and DataTherm

**Figure 3:**
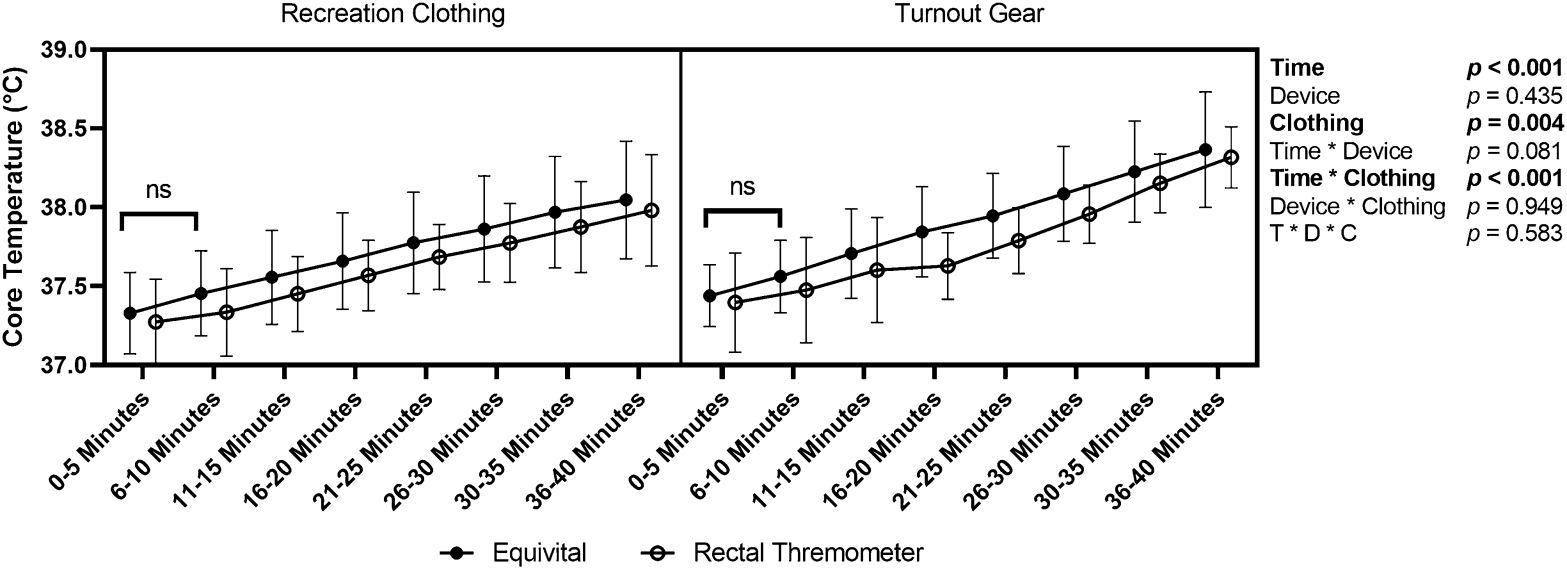
Representation of changes over time in core temperature measurements. Condition (recreational clothing × turnout gear) was used as a covariate. Data represented as mean and standard deviation. Unless specified, all timepoints were significantly different (all *p-*values < 0.037). ns = not significantly different (p > 0.05). Made in Graphpad Prism.

There was a significant effect of time (*p* < 0.001) with core temperature and estimated core temperature increasing with longer exposure to the heat chamber and a significance difference between clothing conditions (*p* = 0.004). However, there was not as significant difference between devices (*p* = 0.435). There was an interaction of time and clothing (*p* < 0.001). No other significant interactions were observed in the mixed effects analysis (*ps* > 0.081). Data is visually represented in Fig. 3.

### Impact of turnout gear on physiological strain and perceived exertion

**Figure 4:**
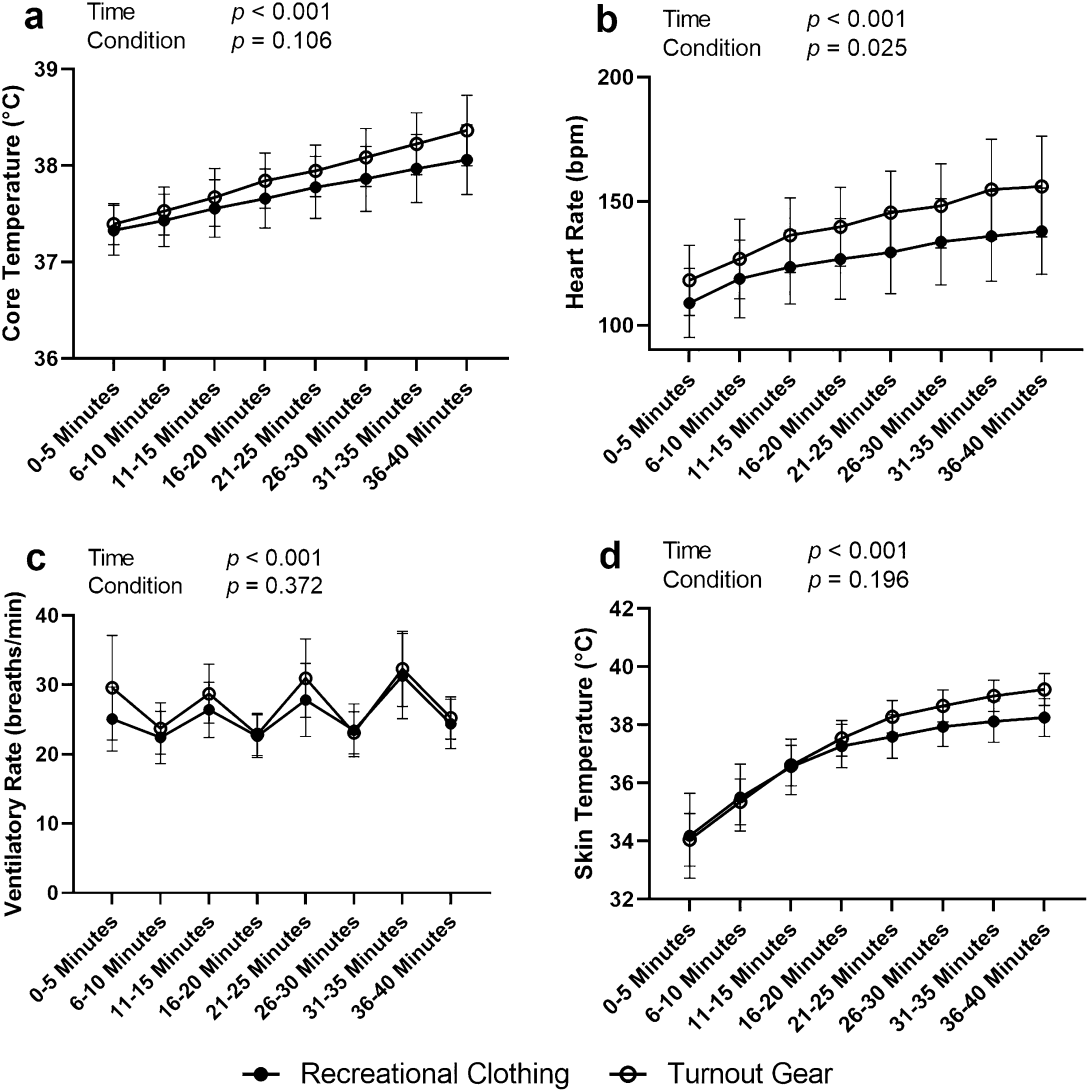
Resulting post hoc ANOVA analyses from MANOVA analysis. Data represented as mean and standard deviation. Effects are reported from the MANOVA test. Made in Graphpad Prism.

The MANOVA included variables collected using the Equivital device (Estimated core temperature, skin temperature, heart rate, and ventilatory rate). Box’s homogeneity of variance was met (*p* = 0.052) and Shapiro Wilk’s multivariate normality was violated (*p* < 0.001), therefore Pillai’s Trace (V) was used for interpretation. Time (V = 1.234, *p* < 0.001) and clothing (V = 0.202, *p* < 0.001) were significant, however no interaction effect was observed (V = 0.140, *p* = 0.911). Univariate ANOVAs were run on significant multivariate statistics with all univariate analyses being significant across time (*ps* < 0.001) and clothing (*ps* < 0.001). Univariate ANOVAs were not run on null multivariate effects. Data is visually represented in Fig. 4.

Regarding pairwise post hoc tests for the Equivital estimated core temperature, there were consistent significant differences across time (*ps* < 0.001) with temperature increasing with heat exposure and no difference between clothing conditions (*p* = 0.107). The pairwise post hoc tests for heart rate revealed all time points were significantly different (*ps* < 0.019) except the following paired timepoints: minutes 11–15 vs minutes 16–20 (*p* = 0.675), minutes 16–20 vs minutes 21–25 (*p* = 0.312), minutes 21–25 vs minutes 26–30 (*p* = 0.410), minutes 26–30 vs minutes 31–35 (*p* = 0.610), and minutes 31–35 vs minutes 36–40 (*p* = 0.609). Interestingly, the nonsignificant timepoints are consecutive timepoints, yet nonconsecutive timepoints remain significant. A significant effect of clothing on heart rate was observed in the pairwise comparisons (*p* = 0.022), with higher heart rates being observed in the firefighter turnout gear than in recreational clothing.

Time had a significant effect on ventilatory rate (*p* < 0.001), however it appears that exercise modality may have been a confounding factor. Participants switched from treadmill walking to deadlifting every 5 min, the same amount of time as our epochs (pairwise comparisons of 5-min epochs can be found in Supplemental Table 1. To eliminate this statistical noise within the analysis we used 10-min epochs for interpretation by averaging consecutive epochs (one treadmill walking and one deadlifting) (Supplemental Table 2). Based on the analysis of 10-min epochs, ventilatory rate in the last 10 min of our exercise protocol was significantly higher than the first three 10-min epochs (*ps* < 0.010). There were no other significant effects by time regarding ventilatory rate. Furthermore, we did not observe a significant effect of clothing on ventilatory rate (*p* = 0.410). Pairwise differences in skin temperature were observed across all time points (*ps* < 0.013), with no effect from clothing (*p* = 0.143).

**Figure 5:**
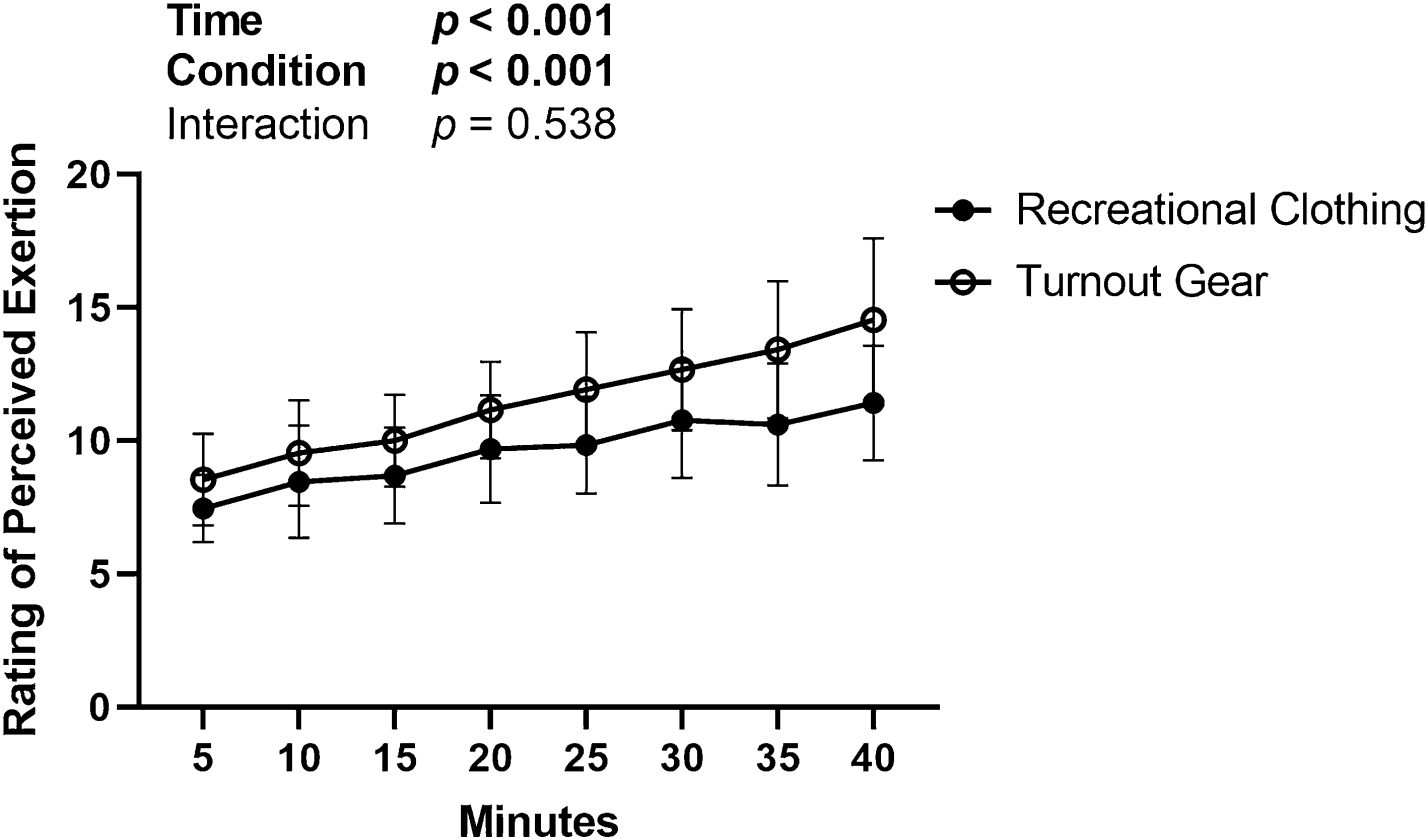
Two-way ANOVA comparison of Rating of Perceived Exertion (RPE) utilizing the Borg 6–20 Scale. Data represented as mean and standard deviation.

The repeated measures ANOVA investigating perception of exertion indicated a significant effect of time (*p* < 0.001) and condition (*p* = 0.010), but no interaction effect of time by condition (*p* = 0.538) for RPE. Pairwise comparisons indicate that RPE was significantly higher minutes for at 16, 21, 26, 30, 35, and 40 min compared to 5 min (ps < 0.001). Additionally, RPE was significantly higher at 25, 30, 35, 40 min compared than at 10 min (ps < 0.040) and 30, 35, 40 were significantly higher than 15 min (ps < 0.002). Lastly, RPE at 20 min and 25 min were significantly higher than at 40 min (ps < 0.016). Data is represented visually in Fig. 5 and average and max values for RPE between conditions are included in Table 1.

**Figure 6:**
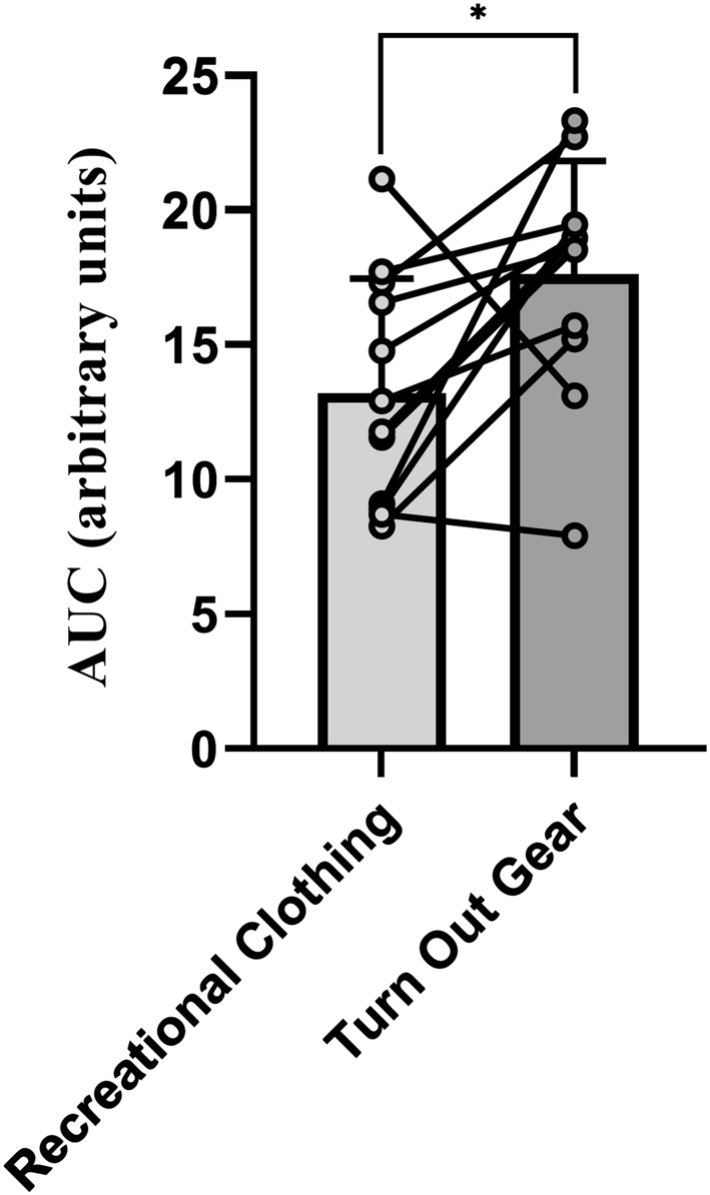
Student *t*-test comparison of cumulative heat stress between conditions. Graph represent mean and standard deviation and individual data points represent individual participants. *Denotes significant difference p < 0.05. Made in Graphpad Prism.

Trapezoidal summation on rectal core temperature was conducted as an exploratory analysis to generate individual area under the curve (AUC) heat responses as a potentially novel method of addressing heat stress, using a *t*-test to compare the differences in heat stress between the clothing condition. AUC was calculated using changes in core temperature over the duration of the exercise protocol. These changes were calculated by subtracting the average of the first 5-min epoch from all succeeding 5-min epochs. The *t*-test concluded there was a significant difference in heat stress as calculated by AUC (*p* = 0.020, *d* = 9.47) (Fig. 6).

## Discussion

This study investigated the validity of the Equivital core temperature monitoring system in comparison to DataTherm II rectal temperature measurements during simulated firefighting in a laboratory setting. In agreement with our primary hypothesis, our results suggest that the Equivital heart rate derived estimation of core temperature provides sufficiently reliable values when compared to DataTherm II rectal core temperatures during simulated firefighting tasks. While the correlation coefficient suggests a moderate-strong relationship between the measures; the two one-sided *t*-tests also suggest the measures are statistically equivalent. The Equivital did estimate a significantly higher average core temperature, however this difference was 0.1 °C, and is supported by 0.09 °C bias observed from the Bland–Altman Plot (Fig. 1). This is known not to represent a clinically relevant difference in core temperature^42^. Our finding of a 0.1 °C difference is substantially smaller than the 0.4 °C difference determined as acceptable in previous research^43^.

Previous research investigating the use of the Equivital algorithm found that the Equivital Black Ghost system underestimated core temperature by ~ 0.75 °C with up to 1.7 °C variation when compared to ingestible core temperature monitoring pills^24^. The divergence between our results and previous work is most likely due to equipment choice and subsequent potential differences in methodology resulting from different devices used (i.e., temperature monitoring pills vs rectal thermometers). Interestingly, the Equivital algorithm was developed using core temperature measures from ingestible pills Our results in combination with a previous validation study^24^ suggest the algorithm may more accurately estimate core temperatures measured via a rectal thermometer, which are a more accurate reflection of true core temperatures^15,16^. In previous investigations, participants completed a variety of tasks at different durations commonly used during firefighter training designed to simulate activities of active fireground conditions. However, recent data indicates that a majority (~ 80%) of firefighters’ calls are non-fireground activities (i.e., highway extractions, and medical calls)^25^. Therefore, the present findings provide generalizable results related to these non-fireground occupational demands.

In contradiction to our second hypothesis, we observed no difference between the Equivital Black Ghost estimated core temperature and DataTherm measures in the rate of core temperature increase during the SFT regardless of time or condition. Core temperature rose at different rates in turnout gear compared to recreational clothing, suggesting greater increases in both core temperature and estimated core temperature with longer heat exposure. However, even though the turnout gear impacts core temperature increases, the agreement between devices was unaffected. These finding suggest that the difference between the Equivital estimated core temperature and rectal thermometer measurements do not significantly diverge during prolonged exercise in hot temperatures. The heart rate-derived algorithm appears to be robust enough to handle this difference without leading to significant differences in core temperature estimates. It is however worth noting, that while not significantly different, as exercise in turnout gear continued the average change in estimated core temperature was larger than the change in rectal core temperature throughout exercise until the final 10 min. This may be due to the slower response time of rectal temperature changes compared to the ingestible pills used to develop the Equivital algorithm. This should be considered for future work investigating whether continued exercise in hot environments with turnout gear would lead to a larger divergence in the estimated core temperatures validity compared to rectal thermometer measures.

Taking the results from our primary and secondary aim together (the agreeability across temperatures, over time, and between clothing conditions) they suggest the Equivital, and black ghost software core temperature algorithm is a consistent and valid estimate of core temperature in research settings and may be an effective tool for core temperature monitoring in field settings such as non-fireground activities. The Equivital did overestimate core temperature by 0.1 °C, however this may add to the utility of the Equivital estimation. The device is not invasive, and the conservative core temperature estimate may result in users reacting to heat stressors before imminent, and potentially irreversible, consequences.

In partial agreement to our tertiary hypothesis, we did see a significant impact of turnout gear on physiological strain. Our multivariate analysis of all Equivital data indicates that these variables differed over time and by clothing condition. Post-hoc univariate analysis of each variable indicates the difference in clothing was driven by heart rate, as this was the only variable significantly affected by turnout gear. This difference in heart rate was likely due to the added external load created by the weight of the turnout gear (~ 20 lbs). As the Black ghost software uses heart rate as a variable of estimating core temperature, the differences in heart rate across condition may explain its ability to overcome core temperature differences and still maintain an accurate assessment of core temperature between clothing conditions; confirmed by the equivalence between Equivital core temperature estimate and DataTherm II rectal core temperature measurement.

We further elucidated the cumulative physiological strain created by firefighter turnout gear by assessing core temperature AUC. This as an assessment of cumulative thermal load, a contributor to increased physiological strain. When comparing AUC between clothing conditions we observed a significant difference in total heat stress in turnout gear. This is in agreement with previous work that indicates turnout gear itself induces a significant increase in physiological strain and cumulative thermal stress^44,45^. This is likely due to the insulative properties of the turnout gear impairing the transfer of heat to the environment. This impaired transfer of heat leads to an increase in body heat storage and contributes to increased thermal stress and physiological strain. In addition, core temperature, heart rate, and skin temperature appear to increase more rapidly in turnout gear, although these were not significant. Investigations using a longer duration and larger sample size may reveal an interaction between gear and duration of heat exposure influencing physiological variables. However, previous research has shown increased physiological strain with both shorter and longer durations of activity involving maximal treadmill testing and simulated firefighting task circuits^46–48^. Conversely, research in wildland firefighters^49^ indicated little impact of full PPE (i.e. boots, helmet, gloves, neck shroud, and googles) on heart rate, oxygen consumption, core temperature, skin temperature, and physiological strain index during the first 60–100 min of treadmill walking^49^. Difference in findings could be related to the weight of full turnout gear which can differ from standard wildland firefighting gear and the selection of tasks used to mimic firefighting activities.

There were no significant differences in physiological strain associated with the gear other than heart rate. Results did indicate perceived exertion was significantly higher and increased more rapidly over time when participants were wearing turnout gear compared to recreational clothing. The relation between heart rate and RPE were expected, as the Borg scale was developed based on heart rate measurements^32^. These findings suggest a possible disproportionate rise in perception of exertion in the absence of increased physiological strain or thermal load due to the turnout gear. This could be due to thermal discomfort from the insulative properties of turnout gear. However, this is speculative and based on previous research that highlighted the perceived thermal discomfort of firefighter gear^50,51^, as we did not assess thermal discomfort in this investigation. Previous research has elucidated negative effects of turnout gear on mobility and performance^11,52^ suggesting limitations in mobility, perceived exertion, and thermal discomfort may impact performance in turnout gear more than actual differences in physiological responses.

The present investigation has demonstrated that the Equivital with Black Ghost software is an accurate, noninvasive, and safe method of estimating core body temperature in healthy young adults. Future investigations should ensure adequate age diversity within their samples to improve the generalizability of these findings considering the diverse age, fitness, and health characteristics of current United States firefighters^53^. In addition, the use of core temperature AUC as a measure of cumulative heat stress may be a valuable workload monitoring tool specifically in firefighters and other outdoor occupational workers that experience multiple bouts or extended heat exposure during their workday. Previous work in underground miners suggest this concept of the dynamic patterns of heat strain over time has a larger impact on heat related injury risk than maximum temperature reached^1,38^. These results in addition to the data presented in the current study warrant future investigation into the implementation of core temperature AUC monitoring.

A potential limitation of our study was researchers did not verify appropriate insertion of the probe prior to beginning the exercise bout and could not control and potential movement of the probe once the exercise bout began. However, participants were given detailed insertion instructions, offered visual assistance of a permanent mark on the probe to ensure full (15 cm) insertion and were provided adhesive tape to secure the probe in place prior to experimental testing. Additionally, while understanding there are limitations to rectal temperature monitoring, it remains the gold standard for core temperature assessment. Ingestible temperature pills also present their own unique problems (ingestion timing, recent food consumption, and hydration). An additional limitation is that while the movements chosen for the SFT were developed in partnership with the local fire department leadership, these movement patterns may not precisely reflect activity required during occupational tasks in real emergency scenarios. However, these movements were chosen as they mimic the most common movement patterns and could be completed in the environmental chamber. The participants in the current investigation were not regular wearers of firefighter gear. This could potentially result in differences in perceived exertion and physiological response (potentially elevated sympathetic response) compared to firefighters who wear this gear on a regular basis., we did not control for circadian rhythm and menstrual cycle in female participants which could influence thermoregulatory response. Participants were encouraged to complete their second visit exactly 1 week after their previous session at the same time of day, however this was not always possible.

Participants in the current study wore turnout gear without boots and a helmet. A 2014 study established component contribution of firefighting gear during 30-min of walking at 5.5 km/h^12^. These findings highlighted that removing standard firefighting boots significantly reduced the physiological strain experienced as boots are mechanically insufficient and restricting heat dissipation from the feet^12^. However, the previously mentioned study in wildland firefighters indicated little impact of full PPE (i.e. boots, helmet, gloves, neck shroud, and goggles)^49^. This may suggest the type of gear worn by wildland firefighters compared to structural firefighters may have a differential impact on physiological strain and heat related injury risk. Future work should consider both the component contribution of gear and outdoor thermoregulatory factors that may impact the validity of this monitoring tool. Additionally, it is important to note that while considered statistically equivalent and a small average bias (0.1 °C), the lower bound suggests notable individual variability with some participants estimated core temperature underestimated by as much as 0.76 °C compared to rectal temperature. This is an important consideration when assessing risk for exertional heat injury. It is important not to only rely on the estimated core temperature, but to additionally monitor other potential symptoms of exertional heat injury (i.e. nausea, dizziness, confusion, etc.).

A strength of the present investigation was purposeful representation of females in the study. Inclusion of females and minorities has been lacking in past research and a call for inclusion has been made by the government, Department of Defense and by the National Health Institute^54^. Another strength of our study was our aim to investigate the validity of the Equivital in non-fireground activities, the environment most commonly experienced by firefighters. Future directions for this work include validating the Equivital in more extreme environments (i.e., structural fires or live-burn training) to determine if the device provides adequate, real-time assessments of high-thermal stress in life-or-death situations. The use of an environmental chamber effectively provided a consistent and reliable control of heat and humidity that is not possible in field-testing environments. Control of these environmental factors reduces confounding variables related to the environment. However, solar radiation and substantial airflow are absent in the environmental chamber; both of which play a role in human thermoregulatory responses and may impact core temperature and heart rate based estimates of core temperature uniquely^55^.

## Conclusions

This study indicates that Equivital EQ02 + LifeMonitor and Black Ghost software provides a reliable, noninvasive, alternative method of continuous core temperature monitoring when performing firefighter occupational tasks with and without turnout gear. Our results support the Equivital as an effective core temperature monitoring system in the laboratory research setting and potentially for non-fireground activities. This study demonstrated that as exercise in heat continues and core temperature rises, the agreement of the Equivital estimated core temperature and DataTherm II rectal core temperature measures remain consistent. It is important to note the individual variability of the estimation and assure that other symptoms of exertional heat injury are monitored in tandem with the estimated core temperature. This study also provides evidence that turnout gear significantly impacts perceived exertion and heart rate, but not other measures of physiological strain (ventilation, core temperature, and skin temperature) experienced during the simulated firefighting activities used in this study, with duration of exertion and heat exposure being more indicative of increases in physiological strain.

### Supplementary Information

Supplementary Table 1.

Supplementary Table 2.

## Data Availability

The datasets generated and analyzed during the current study are available from the corresponding author on reasonable request.
