# Supplementary material for: Validity of heart rate derived core temperature estimation during simulated firefighting tasks
Source: Sci Rep. 2023 Dec 15;13:22503. doi: 10.1038/s41598-023-49929-x (PMC10728086; doi:10.1038/s41598-023-49929-x)
Supplement: Supplementary file 1 — Supplementary Table 1. [file 41598_2023_49929_MOESM1_ESM.docx]

| **Supplemental Table 1. Pairwise Post Hoc Comparisons (5-minute epochs)** | | | | | | |
| --- | --- | --- | --- | --- | --- | --- |
| Pairwise Comparisons (Time) | | | Equivital Core Temperature | Heart Rate | Ventilatory Rate | Skin Temperature |
| Minutes 1-5 | - | Minutes 6-10 | **< 0.001** | **0.010** | **0.012** | **< 0.001** |
|  | - | Minutes 11-15 | **< 0.001** | **< 0.001** | 1.000 | **< 0.001** |
|  | - | Minutes 16-20 | **< 0.001** | **< 0.001** | **0.024** | **< 0.001** |
|  | - | Minutes 21-25 | **< 0.001** | **< 0.001** | 0.943 | **< 0.001** |
|  | - | Minutes 26-30 | **< 0.001** | **< 0.001** | **0.049** | **< 0.001** |
|  | - | Minutes 31-35 | **< 0.001** | **< 0.001** | 0.050 | **< 0.001** |
|  | - | Minutes 36-40 | **< 0.001** | **< 0.001** | 0.140 | **< 0.001** |
|  |  |  |  |  |  |  |
| Minutes 6-10 | - | Minutes 11-15 | **< 0.001** | **0.019** | **< 0.001** | **< 0.001** |
|  | - | Minutes 16-20 | **< 0.001** | **< 0.001** | 0.991 | **< 0.001** |
|  | - | Minutes 21-25 | **< 0.001** | **0.001** | **< 0.001** | **< 0.001** |
|  | - | Minutes 26-30 | **< 0.001** | **< 0.001** | 1.000 | **< 0.001** |
|  | - | Minutes 31-35 | **< 0.001** | **< 0.001** | **< 0.001** | **< 0.001** |
|  | - | Minutes 36-40 | **< 0.001** | **< 0.001** | 0.240 | **< 0.001** |
|  |  |  |  |  |  |  |
| Minutes 11-15 | - | Minutes 16-20 | **< 0.001** | 0.675 | **< 0.001** | **< 0.001** |
|  | - | Minutes 21-25 | **< 0.001** | **0.002** | 0.963 | **< 0.001** |
|  | - | Minutes 26-30 | **< 0.001** | **0.004** | **< 0.001** | **< 0.001** |
|  | - | Minutes 31-35 | **< 0.001** | **< 0.001** | 0.103 | **< 0.001** |
|  | - | Minutes 36-40 | **< 0.001** | **< 0.001** | **0.008** | **< 0.001** |
|  |  |  |  |  |  |  |
| Minutes 16-20 | - | Minutes 21-25 | **< 0.001** | 0.312 | **0.001** | **< 0.001** |
|  | - | Minutes 26-30 | **< 0.001** | **0.001** | 0.914 | **< 0.001** |
|  | - | Minutes 31-35 | **< 0.001** | **0.019** | **< 0.001** | **< 0.001** |
|  | - | Minutes 36-40 | **< 0.001** | **< 0.001** | 0.049 | **< 0.001** |
|  |  |  |  |  |  |  |
| Minutes 21-25 | - | Minutes 26-30 | **< 0.001** | 0.410 | **0.008** | **< 0.001** |
|  | - | Minutes 31-35 | **< 0.001** | **0.002** | 0.290 | **< 0.001** |
|  | - | Minutes 36-40 | **< 0.001** | **0.002** | **0.049** | **< 0.001** |
|  |  |  |  |  |  |  |
| Minutes 26-30 | - | Minutes 31-35 | **< 0.001** | 0.61 | **< 0.001** | **0.003** |
|  | - | Minutes 36-40 | **< 0.001** | **0.001** | 0.288 | **< 0.001** |
|  |  |  |  |  |  |  |
| Minutes 31-35 | - | Minutes 36-40 | **< 0.001** | 0.609 | **< 0.001** | **0.003** |
|  |  |  |  |  |  |  |
| Time |  |  | **<0.001** | **<0.001** | **<0.001** | **0.015** |
| Condition |  |  | 0.107 | **0.022** | 0.410 | 0.143 |
| Pairwise post hoc comparisons from two-way MANOVA across all Equivital variables between conditions (recreational clothing x turn out gear). Presented as the *p*- values for pairwise comparisons. Significant *p*- values have been bolded. | | | | | | |
