# Supplementary material for: Validity of heart rate derived core temperature estimation during simulated firefighting tasks
Source: Sci Rep. 2023 Dec 15;13:22503. doi: 10.1038/s41598-023-49929-x (PMC10728086; doi:10.1038/s41598-023-49929-x)
Supplement: Supplementary file 2 — Supplementary Table 2. [file 41598_2023_49929_MOESM2_ESM.docx]

x

| **Supplemental Table 2. Ventilatory Rate Pairwise Comparisons (10-minute epochs)** | | | |
| --- | --- | --- | --- |
| Pairwise Comparisons (Time) | | | *p*-value |
| Minutes 1 – 10 | - | Minutes 11 - 20 | 0.987 |
|  | - | Minutes 21 - 30 | 0.633 |
|  | - | Minutes 31 - 40 | **0.002** |
| Minutes 11 - 20 | - | Minutes 21 - 30 | 0.484 |
|  | - | Minutes 31 - 40 | **0.009** |
| Minutes 21 - 30 | - | Minutes 31 - 40 | **0.010** |
| Condition (Clothing) | | | 0.410 |
